# Mapping the oral resistome: a systematic review

**DOI:** 10.1099/jmm.0.001866

**Published:** 2024-08-12

**Authors:** Smitha Sukumar, Zalmay Rahmanyar, Hagaar Q. El Jurf, William S. Akil, Jafar Hussain, F. Elizabeth Martin, Kanchana Ekanayake, Elena Martinez

**Affiliations:** 1 Faculty of Medicine and Health, The University of Sydney, Sydney, New South Wales, 2000, Australia; 2 Institute of Clinical Pathology and Medical Research, Westmead Hospital, Westmead, New South Wales, 2145, Australia

**Keywords:** antimicrobial resistance, antimicrobial resistance genes, oral cavity, resistome, systematic review

## Abstract

Studying individual ecological niches within the oral cavity is a logical first step to understanding the distribution of antimicrobial resistance genes (ARGs); however, it is not representative of the whole oral resistome. The aim of our systematic review was to provide a map of the oral resistome by reviewing the composition of individual niches. A total of 580 papers were retrieved from a search of all English language publications investigating the presence of oral ARGs in five electronic databases between January 2015 and August 2023. Fifteen studies [10 PCR and 5 next-generation sequencing (NGS)] were included in this review. The heterogeneity of methods precluded meta-analysis. ARGs are present throughout the oral cavity with 158 unique ARGs identified across 6 locations – supra and sub-gingival biofilm, mucosa, oropharynx, root canal system (RCS) and saliva. The supragingival biofilm had the highest resistome richness, while the RCS had the least. Tetracycline was the dominant antimicrobial resistance (AMR) class found. Three core genes were identified – *tet(M*), *tet(O*) and *ermB*.This review highlights the necessity of NGS studies to comprehensively characterize the oral resistome in its entirety. This is the logical foundation for future ‘omics studies to truly understand the scope of the resistome and its contribution to AMR.

## Introduction

The discovery of antimicrobials was a major medical milestone that has saved and continues to save millions of lives every year. Antimicrobials have a range of clinical indications, including treating potentially fatal infections such as pneumonia, septicemia and meningitis. In dentistry, antimicrobial agents are generally used as an adjunct to surgical therapy for odontogenic infections and are prescribed prophylactically for at-risk patients to prevent life-threatening infections prior to specified dental treatments [1]. Worldwide, 3–11% of all antibiotic prescriptions are written by dentists [2], who most commonly prescribe beta-lactams, followed by nitroimidazoles, lincosamides, macrolides and tetracyclines [3].

The therapeutic effectiveness of antibiotics is threatened by the ability of bacteria to adapt to and resist the actions of these drugs. Antimicrobial resistance (AMR) has rapidly become an international threat to public health driven by the misuse and overuse of antibiotics with up to 50% of all antibiotics prescribed worldwide deemed unnecessary [4]. In Australia, 80% of antibiotics prescribed by dentists were inappropriate (do not follow national guidelines) [5]. These prescribing patterns have resulted in increased patient morbidity and mortality, longer hospital stays and higher healthcare costs as bacterial infections become harder to treat [6]. In 2019, over 6 million deaths were estimated to be associated/attributable to bacterial AMR [7]. By 2050, the World Bank projects that AMR will cost the global economy between USD 300 billion and more than USD 1 trillion annually [8].

Resistance is commonly acquired via antimicrobial resistance genes (ARGs) and is spread through bacterial communities (within and to different species) by horizontal gene transfer [9]. Resistance genes are found in diverse human, animal and environmental microbial communities. These collections of ARGs are known as resistomes, and the human oral cavity, the second largest microbiome in the body, is a known reservoir of potentially transferrable ARGs [10].

Our understanding of the oral resistome is rapidly expanding and reflects significant advances in molecular microbiological methodologies [11]. The use of PCR in direct samples eliminated the need for culture (with its limitations) and allowed the identification of ARGs within microbial communities. The advent of next-generation sequencing (NGS) and the use of metagenomics have further revealed the scale and complexity of the oral resistome. However, studies to date focus on profiling the resistome of particular ecological niches within the oral cavity.

The mouth is a unique environment containing both hard and soft tissue surfaces lubricated by saliva. This creates different ecological niches, ranging from biofilms on hard surfaces like the tooth/dental plaque interface to planktonic bacteria within saliva. The oral cavity is a dynamic environment. It undergoes significant disruption every day during eating or tooth brushing and throughout life, either physiologically (eruption of teeth) or pathologically (development of dental caries or periodontal disease), which impacts the bacteria present and the ARGs they carry. Single-niche resistome profiling provides insights into diseases where the interaction of a particular bacterial community, e.g. the dental biofilm beneath the gums (subgingival biofilm) drives periodontal disease. However, this is not representative of the oral resistome as a whole.

A comprehensive understanding of the oral resistome provides information about (1) which ARGs can be most commonly found; (2) their potential for mobilization and/or transfer between different organisms found in the oral microbiota and (3) distant transmission of AMR via oral bacteria, which can move into the systemic circulation (transient bacteremia caused by oral hygiene practices) and to others (breathing and kissing) [12]. More importantly, a comprehensive profile of the oral resistome provides a powerful educational tool to inform antimicrobial stewardship programmes for healthcare professionals and the wider community to promote the judicious use of antibiotics.

The aim of this systematic review is to provide a map of the oral resistome by reviewing the composition of individual niches and identifying a core or ‘global’ oral resistome.

## Methodology

The search strategy for peer-reviewed literature was developed in consultation with an expert medicine and health sciences librarian (KE) from The University of Sydney. The following electronic databases were searched for relevant peer-reviewed literature between January 2015 and 12 August 2023 – MEDLINE (Ovid SP), Embase (Ovid SP), Web of Science (Clarivate), CINAHL (EBSCO Host) and Scopus (Elsevier). The search strategies used for all five databases are presented in the supplementary figure. This specific timeframe was chosen based on the publication of the last systematic review, with a focus on identifying studies utilizing the most advanced molecular microbiological techniques.

**Fig. 1.**
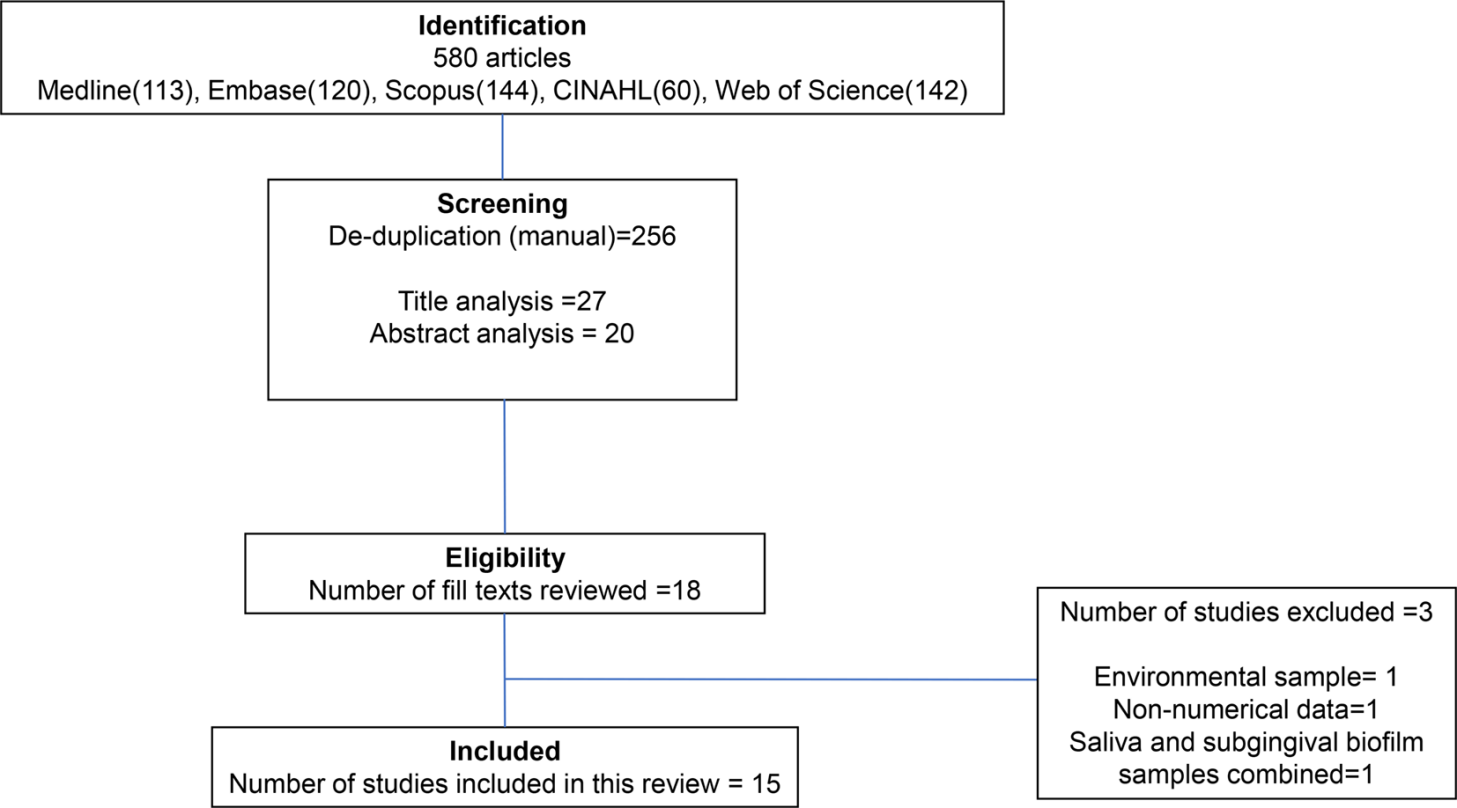
Study selection process (adapted from the Preferred Reporting Items for Systematic Reviews and Meta-Analyses flow diagram) undertaken for this systematic review.

All records identified from the databases were exported into EndNote 20 reference management software and imported to Covidence systematic review management software (Veritas Health Innovation Victoria, Australia). Once the duplicates were removed, five reviewers (ZR, HQQE, WSA, JH and SS) independently screened the titles and abstracts of the identified studies according to the inclusion criteria. Studies that appeared to meet the inclusion criteria (Table 1), as well as those that required further assessment were retrieved in full text. Full-text reports were independently assessed for eligibility by three reviewers (ZR, HQQE and SS) and the reasons for exclusion of all studies were noted. Any disagreements were resolved through discussions with a fourth reviewer. The results from the search strategy are shown in Fig. 1.

**Table 1.** Study selection criteria

Inclusion criteria	Exclusion criteria
Clinical studies in healthy patients or in patients with oral disease or medical co-morbidities (cross-sectional or longitudinal studies). Studies that detected bacterial resistance genes of antibiotics by molecular techniques, specifically PCR and metagenomics. Studies with samples collected from the oral cavity including saliva, supra or sub-gingival biofilm or the RCS including the pulp and oral mucosa.	Literature reviews *In vitro* studies Studies in animal populations Studies that used mixed samples from the oral cavity Studies that did not use molecular methods for the detection of ARGs Studies in which the objective was not the detection of resistance genes Biofilm studies Lack of data

Variables extracted from the studies include author details, publication year, molecular methodology, number of ARGs detected, health status, sample size, country, age, antibiotic exposure and sample location (Table 2).

**Table 2.** Characteristics of studies included in this systematic review

Authors	Year	Method	ARGs	Health status	*N*	Country	Age in years (mean)	Last dose of antibiotics (months)	ARG location
**Moraes *et al.***[Bibr R25][25]	2015	PCR	1	Healthy endo	42	Brazil	39	3	Saliva supragingival biofilm RCS
**Palma *et al.***[Bibr R18][18]	2016	PCR/NGS	20	Healthy	22	Brazil	0.75	3	Mucosa
**Anderson *et al.***[Bibr R22][22]	2017	PCR	2	Endo	20	Germany	18+	12	RCS
**Gomez-Arango *et al.***[Bibr R17][17]	2017	PCR	1	Healthy	36	Australia	Neonates	+/- intrapartum	Mucosa
**de Lima *et al.***[Bibr R26][26]	2018	PCR	1	Endo	27	Brazil	6	Not stated	Saliva supragingival biofilm RCS
**Guo *et al.***[Bibr R19][19]	2018	PCR	6	Group B strep positive	24	China	Neonates	+/- intrapartum	Mucosa
**Subramaniam *et al.***[Bibr R24][24]	2019	PCR	4	Oral cancer	24	India	47	Not stated	Saliva
**Zhang *et al.***[Bibr R41][41]	2019	NGS	7	Caries	Not stated	China	Not stated	Not stated	Saliva
**Carr *et al.***[Bibr R15][15]	2020	NGS	70	Healthy	351	China, Fiji, Philippines, France, Germany and USA	Not stated	Not stated	Saliva supragingival
**Le *et al.*** [23]	2020	PCR/NGS	13	Medical co-morbidities	98	Japan	83	Not stated	Oropharynx
**Milanovic *et al.***[Bibr R31][31]	2020	PCR	12	Healthy	144	Italy	38	3	Saliva
**Arredondo *et al.*** [16]	2021	PCR	10	Healthy perio	259	Spain	36	3	Sub-gingival biofilm
**Anderson *et al.*** [14]	2023	NGS	15	Healthy caries perio	179	Germany	37	12	Supragingival biofilm
**Sukumar *et al.*** [13]	2023	NGS	60	Healthy	221	Australia	Longitudinal	3	Supragingival biofilm
**Gager *et al.*** [42]	2023	NGS	18	Perio	39	Germany	Not stated	Not stated	Sub-gingival biofilm

ARGs column indicates the number of ARGs identified in the study. Metagenomic studies are denoted as NGS. Health status variables include endo, which is endodontic disease (infection of the pulp and RCS) and Perio, which is periodontal disease (gums and bone).

**Fig. 2.**
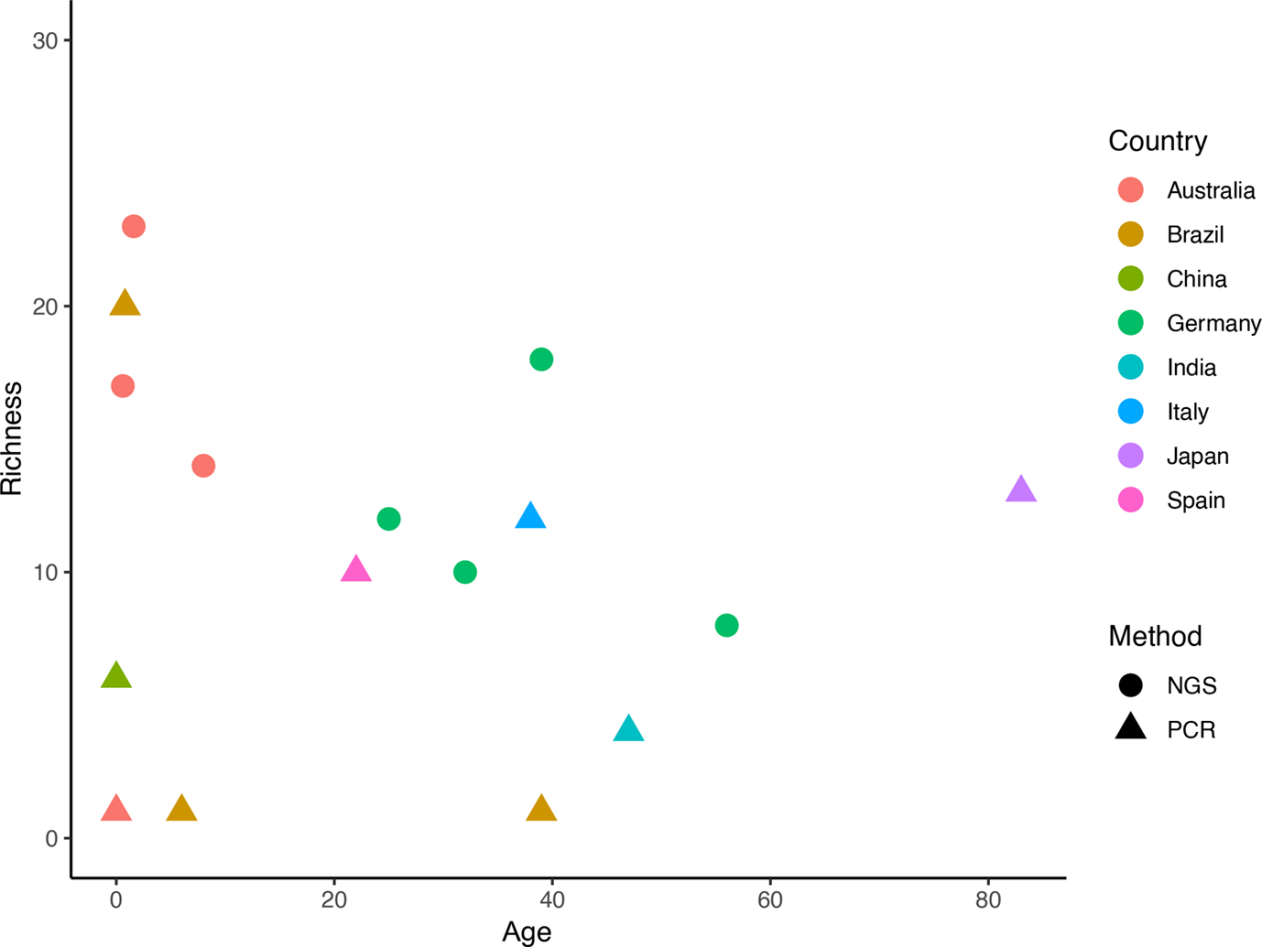
Scatter plot highlighting the heterogeneity of the sample population by age, geographic location and identification method used, which in this review was PCR or metagenomics which is denoted here as NGS.

Due to methodologic differences among the studies (Fig. 2), it was not possible to perform a meta-analysis.

## Results

In total, 15 studies met the inclusion criteria for this systematic review (Table 2) with 67% of the studies being PCR-based. The remaining five studies utilized NGS (short read sequence-based metagenomics) with three published in 2023.

The studies surveyed populations from 12 countries around the world with an age range spanning from neonates to older adults (101 years). Thirty-three per cent of studies investigated a paediatric population over the first decade of life. The study populations ranged from exclusively healthy participants or those with oral diseases and systemic illnesses or combined populations (Table 2).

PCR-based studies identified an average of seven ARGs (range: 1–20) compared to 34 (range: 7–70) ARGs identified by metagenomic studies. Overall, 159 genes resistant to 22 antibiotic classes were found in six locations in the oral cavity – dental biofilm (sub- and supragingival), oral mucosa, saliva, RCS and the oropharynx in health and disease (Table S1a and b, available in the online version of this article).

### Resistome composition varies by ecological niche

**Fig. 3.**
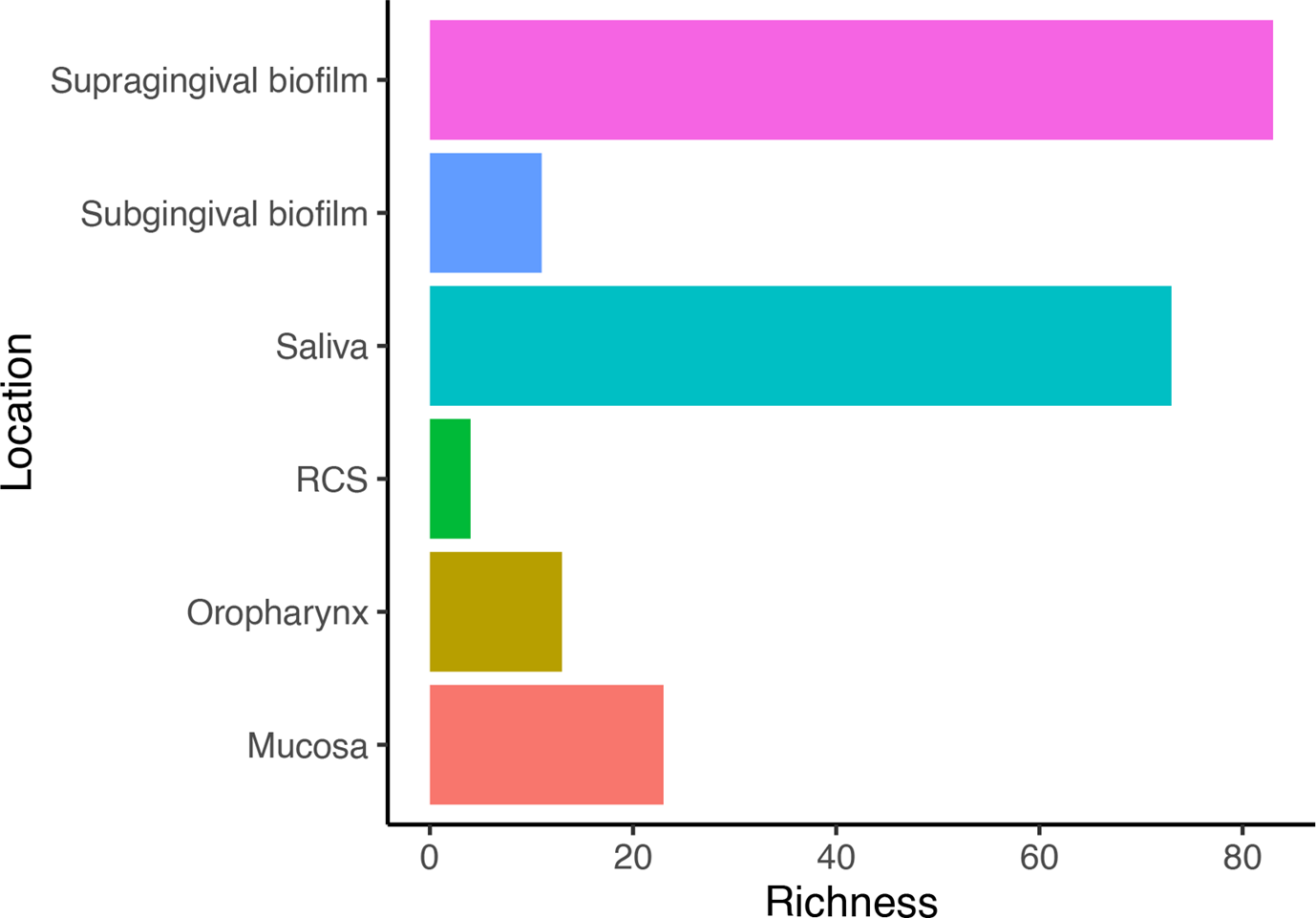
Number of unique ARGs (richness) identified from the six oral ecological niches reviewed including the RCS.

**Fig. 4.**
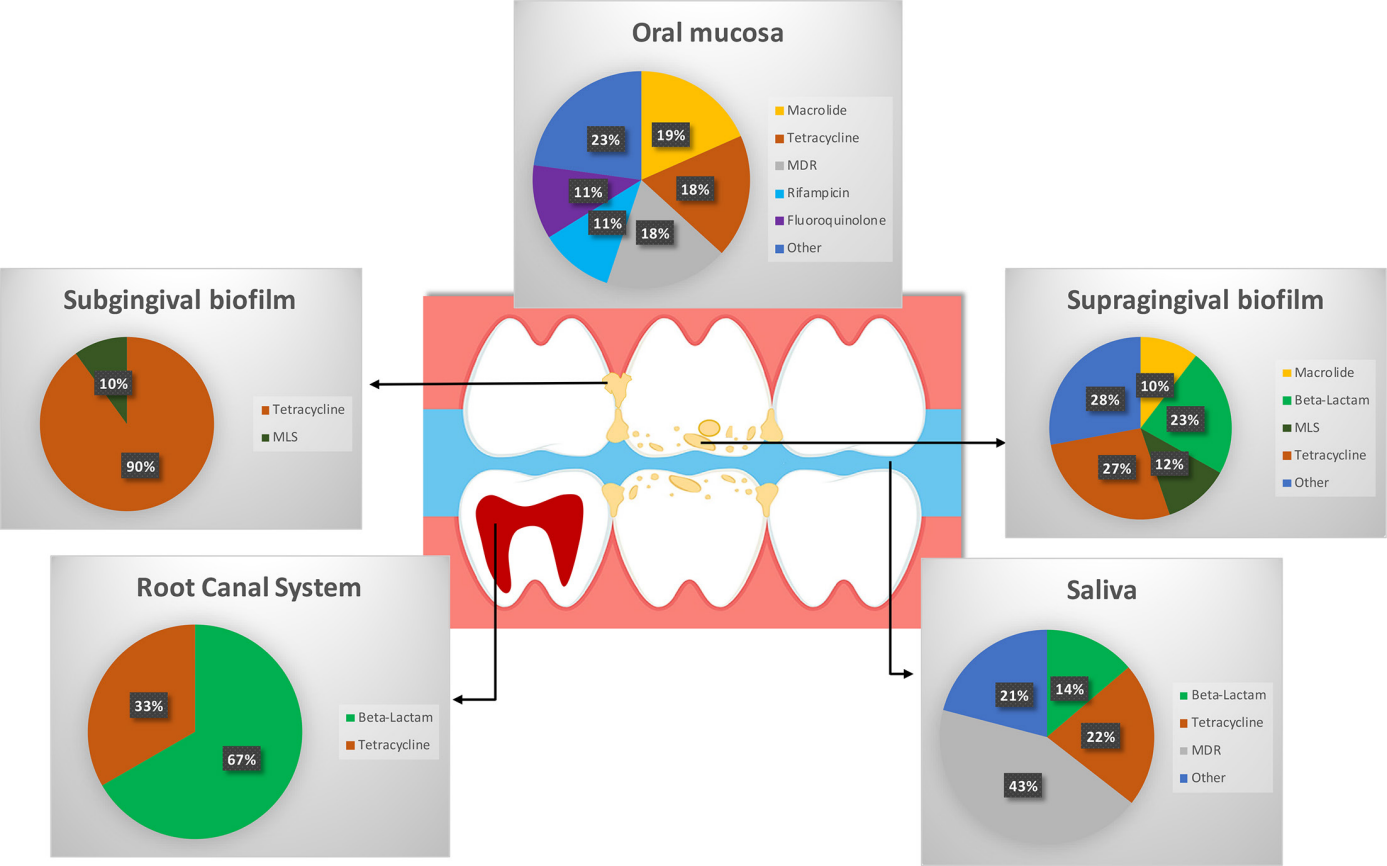
Resistome map showing the distribution of ARGs by class of antibiotic within each oral ecological niche. MDR, multidrug resistance; MLS, macrolide/lincosamide/streptogramin.

Resistome composition is described in terms of richness. This is defined as the number of unique ARGs found in each location. Resistome composition varied between locations (Fig. 3). Supragingival biofilm had the most diverse composition, while only four ARGs were identified in the RCS. This finding is mirrored at an AMR class level, with genes resistant to 19 AMR classes present in supragingival biofilm, followed by the mucosa with 10 classes (Fig. 4).

### Three genes were found in all ecological niches

Tetracycline resistance genes were found across all locations, contributing between 18% (mucosa) and 90% (subgingival biofilm) of the oral resistome. This was followed by genes resistant to beta-lactams, which contributed to 14, 23 and 67% of the salivary, supragingival biofilm and RCS resistomes, respectively.

Shared ARGs were analysed across four sites – oral mucosa, supra and sub-gingival biofilm and saliva due to their anatomical proximity and role in oral disease. Forty-nine genes were found in one or more locations. Three genes, the tetracycline resistance genes *tet(M)*, *tet(O)* and macrolide-resistant gene *ermB* were found in all four locations and are defined as core genes. The niches with the highest number of shared ARGs (15) were supragingival biofilm and saliva [*mefA, cfxA, patA, bla-TEM-1, cfxA3, RlmA(II), tetA(60), cfxA2, dfrF, tet40, acrD, acrF, ermA, bacA* and *cfxA6*]. The next highest shared resistome (three common ARGs) was between saliva and mucosa [*mef(A), tet(L), mdtG* and *msbA*] and supra and sub-gingival plaque sharing [*tet32, tet(Q)* and *tet(B)*] (Table S2).

### ARGs were associated with ESKAPE and distant-site pathogens

Most studies (13 of 15) in this review reported on species associated with ARGs. In total, 98 bacteria at a genus level were associated with ARGs from all 6 ecological niches (Table S3). The mean number of bacteria associated with ARGs identified in the three NGS studies [13,15] was 65, compared to an average of 4 ARG-carrying bacteria in 10 PCR studies. At a species level, 25 ARG-carrying species were identified from 10 PCR studies with 177 species/subspecies identified in the NGS studies. While no single genus dominated, 20% of ARGs were associated with streptococcal species, an oral commensal species, followed by *Prevotella* species. ARGs carried on streptococcal species were found in both adults [14,16] and children [13,17,19] in the mucosa and in supra and sub-gingival plaque. Notably, four studies identified ARGs associated with streptococcal species (*Streptococcus sanguinis, mitis and anginosus*) implicated in distant site infections such as infective endocarditis [20]. These distant site pathogens were identified at a species level by PCR and NGS studies [14,16] and at a subspecies level [*S. mitis B6 (taxid 365659)*, *S. anginosus sub*sp. *whileyi MAS624 (taxid 1353243)* and *S. sanguinis (taxid 1305)*] [13].

Other clinically significant organisms identified were the ESKAPE pathogens – *Enterococcus faecium*, *Staphylococcus aureus*, *Klebsiella pneumoniae*, *Acinetobacter baumannii*, *Pseudomonas aeruginosa* and *Enterobacter* spp. These multidrug-resistant bacteria are a major cause of life-threatening nosocomial infections [21]. All six pathogens were identified by PCR and NGS studies to be ARG-carrying bacteria in different oral ecological niches. PCR studies identified *E. faecium* [22]*, A. baumannii* [23] as well as *S. aureus* and *K. pneumoniae* [24]. While metagenomics sequencing revealed ARGs were associated with *E. faecium* [15], *P. aeruginosa* [14] and five *Enterobacter* spp*.* [13].

### Resistome richness decreased in disease states across the oral cavity

We also compared resistome profiles in disease and health based on ecological niche. The criteria set for this analysis were based on the underlying pathophysiology of the diseases. Supragingival biofilm drives the dental caries process, which if left untreated leads to infection of the pulp and RCS (endodontic disease). Supragingival biofilm also drives changes in gingiva and bone (periodontal diseases). Saliva is an important modifier/risk factor in the caries process and lubricates all surfaces in the mouth and thus potentially reflects malignant changes in the mucosa (oral cancer).

**Fig. 5.**
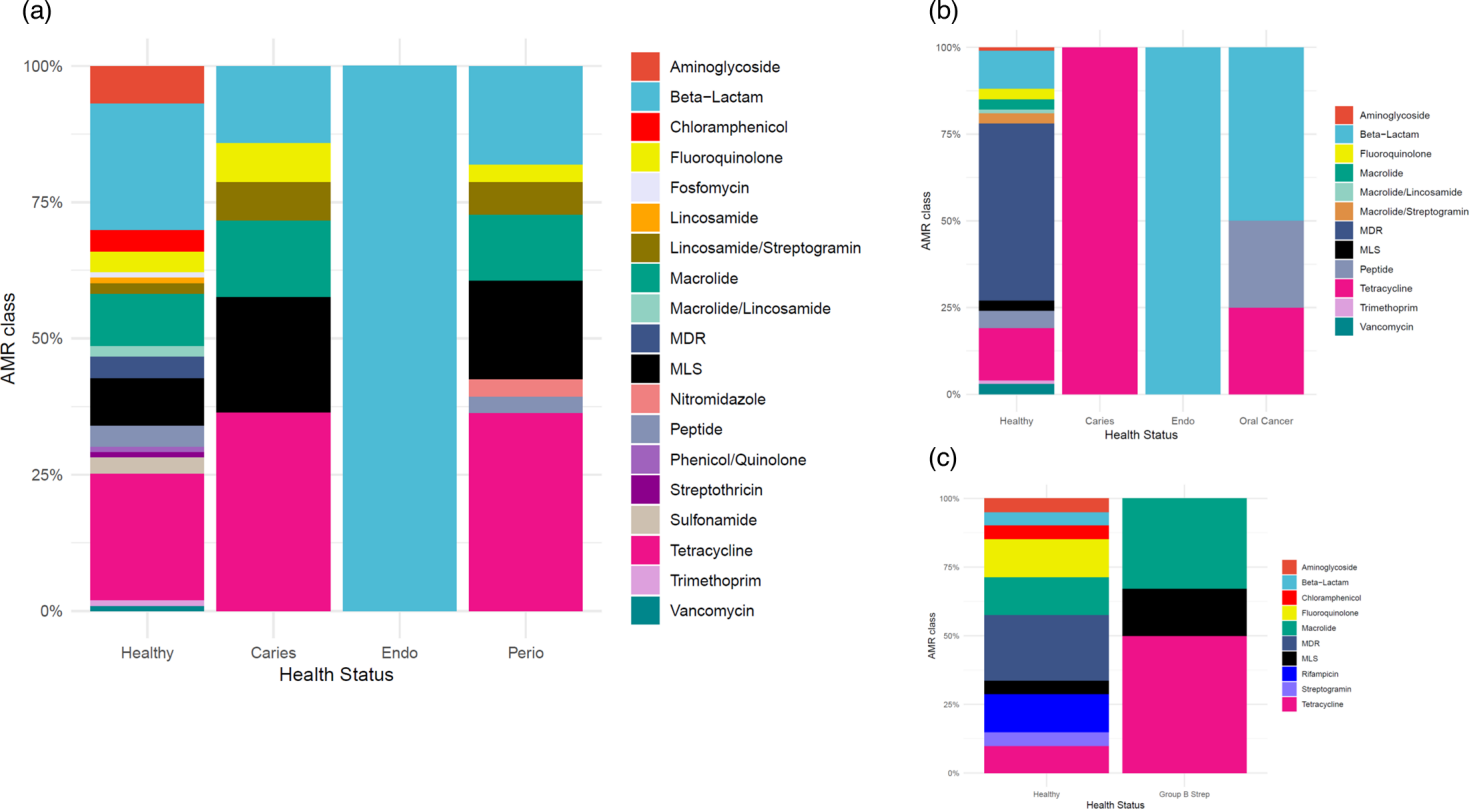
Composition of oral resistome in health and disease in A: supragingival biofilm, B: saliva and C: mucosa. This profile is at the AMR class level. MDR, multi drug resistance; MLS, macrolide/lincosamide/streptogramin.

A consistent trend across the three locations was that a healthy resistome was more diverse than the diseased resistome at an AMR class level (Fig. 5). Consistent with our previous findings, the supragingival resistome (Fig. 5a) had the richest composition across both healthy and diseased states compared to the saliva (Fig. 5b) and the oral mucosa (Fig. 5c). While tetracycline ARGs dominated the supragingival and saliva resistome profiles in caries, the supragingival resistome was richer (six AMR classes) than the salivary resistome profile (tetracycline resistance genes only).

Beta-lactam resistance genes were present in all resistomes when categorized by location or health status with two exceptions – the salivary resistome in caries-affected people and the mucosal resistome in the group B streptococcus infection population. Resistomes in all locations for diseased populations had a greater presence of tetracycline resistance genes – 35% and 36% of the supragingival resistome in caries and periodontal disease, respectively; 100% and 25% of the salivary resistome in caries and oral cancer, respectively; 50% of the mucosal resistome in the group B streptococcus infection population (Fig. 5).

## Discussion

The findings of our systematic review are consistent with the results of the last review in 2015 [25], which confirm the presence of ARGs in all ecological niches in the oral cavity. The inclusion of NGS studies in our search strategy provided a more comprehensive map of the oral resistome with 159 ARGs identified compared to 33 ARGs in the study by Moraes *et al*. [25] with composition varying based on sample site.

The most diverse resistome profile was the supragingival biofilm, which drew on data from two metagenomics studies [13,15]. Metagenomics or shot-gun sequencing is a non-targeted, comprehensive approach used to identify all genes in all organisms present in any given sample compared to PCR, which uses primers targeting known ARGs. This is demonstrated in studies identifying ARGs in RCS. Resistome profiling for this niche is currently impossible, as in this review results were pooled from two PCR [22,26] studies that used primers targeting beta-lactam ARGs only. This severely underestimates the richness of the RCS.

The variation in resistome composition may also be explained by the fact that resistome composition is a reflection of microbiome composition [13]. The oral cavity contains distinct topographies each with unique nutrient gradients, resulting in distinct microbial communities with resistome compositions to match. In this review, the analysis of ARG-carrying species was limited to a qualitative analysis as most studies used PCR for gene detection. A limitation of this method is the selection of appropriate primers, which is contingent on the identification of the species being used as a DNA template [27]. Additionally, the lack of standardization in bioinformatic processing precluded statistical analysis between the metagenomic studies. However, even this qualitative analysis is valuable in highlighting the superiority of metagenomics. Using this platform, 96% of ARG-carrying bacteria were identified at a species or subspecies level compared to 49% in PCR studies (Table S3).

The overarching aim of resistome surveillance studies is to elucidate reservoirs and dissemination routes for AMR. Central to this is the concept of a stable population of highly prevalent ARGs (a core resistome). In this review, we found genes resistant to tetracycline (AMR class level) were present in all five oral niches (Fig. 4). Healthy resistomes in supragingival biofilm, saliva and the oral mucosa had nine AMR classes in common including genes resistant to macrolides, beta-lactams and tetracyclines (Fig. 5). At the gene level, three core ARGs [*tet(M)*, *tet(O)* and *ermB*] were found in all locations, irrespective of health status. Our findings at a gene level (two out of three core genes are resistant to tetracycline) mirror our findings at an AMR class level. The ubiquity of tetracycline ARGs and their presence in paediatric populations including the group B strep positive group is notable, as this antibiotic is not prescribed to children under 12 years old due to its ability to incorporate and stain mineralizing tissue including teeth [28]. These genes may be present due to their ability to be co-carried with another ARG associated with a more commonly prescribed antibiotic such as the macrolide resistance gene *ermB*. Co-carriage is a common feature in the oral cavity facilitated by mobile genetic elements such as the *Tn916* family, which facilitate the movement of *ermB* and *tet(M)* [29].

The dominance of tetracycline resistance in the oral resistome may also be explained by indirect antibiotic exposures such as through diet. While tetracycline is widely used in global food production [30], there is limited evidence about the association between diet and resistome composition, with only one study finding a correlation between dairy intake and the abundance of *tet(K)* and *erm(C)* genes [31].

All the PCR studies in this review targeted tetracycline genes, which may also contribute to the overrepresentation of this AMR class in the profiling.

There were significant limitations in this study; the disparity in data output between PCR and metagenomics resulted in an analysis limited to a single standardized metric (richness) being used to compare the studies. This heterogeneity in methodology was further compounded within each study type; e.g. in PCR studies, the DNA templates ranged from single species [18,22, 25, 26] to swabs from an ecological niche [16,17, 19, 23, 24, 31]. Different primers were used in different studies to identify the same gene, e.g. *tet(M)* [16,18, 22, 31], and two studies utilized NGS platforms to identify ARGs in specific species of interest in addition to PCR identification [18,23] (Table S4). Lack of standardization in metagenomic studies precluded the use of metrics such as diversity or abundance of specific ARGs or ARG-carrying species. For example, relative abundance for supragingival biofilms was calculated using reads per kilobase million in Carr *et al.* [15], while Sukumar *et al.* used transcripts per kilobase million [13].

Genomic surveillance of AMR requires the requisite databases to identify the ARG content of microbial communities. These databases are categorized as either species-specific or mechanism-based, with some focused on a particular resistance mechanism (mutation or acquired), while others cover both [32]. Appropriate selection of the ARG database is critical, as the number of ARGs identified in a surveillance study is key to understanding the true resistance burden in any given community. Four of the five metagenomics studies in this review used the Comprehensive Antimicrobial Resistance Database (CARD) [33], a curated database covering both types of resistance mechanisms. Two studies utilized the ensemble platform ABRicate [34] which assembles data from nine independent annotation tools (species-specific and mechanism-based) including CARD. This casts the widest possible net to identify ARGs and increases accuracy as it allows for cross-referencing. Notably, Anderson *et al.* [14] used ARG-ANNOT [35], a database that has not been updated since 2019 and has been archived. Therefore, the selection of the correct annotation tool has a significant impact on the diversity of ARGs identified. This once again highlights the need for a standardized bioinformatics pipeline for ARG identification or at a minimum defined parameters and metrics to facilitate meaningful comparisons between studies.

Finally, while the combined study population provides a global picture of the resistome in terms of geographic location, age and health and disease, the heterogeneity of the population makes comparisons difficult. A major issue was the lack of consistent metadata collected from participants in many studies. Participant age was not specified in four studies, and only 47% of studies stated the time period since the last course of antibiotics, which ranged between 1 and 12 months. To date, there is a single metagenomic study (*n* = 1) reporting that while oral resistome is affected by direct antibiotic exposure, it demonstrates temporal stability and resilience [36]. Therefore, the effect of previous direct antibiotic exposure remains an important variable to consider when undertaking surveillance studies.

This review once again focuses on the bias of microbiome research, which remains confined to populations in highly developed nations [37]. Three of the four metagenomic studies were done in Germany (*n* = 2) and Australia. Both of these countries have a significantly larger presence in publicly available microbiome databases compared to their population sizes. Australia/New Zealand have the most lopsided presence, accounting for 3.1% of samples collected from only 0.4% of the global population [38]. Carr *et al.* used samples from a range of countries including underrepresented nations such as Fiji and the Philippines; however, the samples were combined, thus precluding geographic profiling [15]. This geographical imbalance has significant clinical consequences, as the fight to curb AMR is a global one. Genetic level surveillance studies of the oral resistome are required for 85% of the population living outside Europe and Northern America to ensure we know the full extent of AMR.

## Conclusion

We analysed 15 studies to compile a resistome map of the oral cavity. While there was variation between sites, tetracycline resistant genes dominated the profiles. Three ARGs were identified in all sites, suggesting the presence of a ‘core’ or global resistome. Our review highlights that the current evidence base is inadequate to comprehensively map the oral resistome. PCR-based studies have no utility in answering ‘what is there?’ and ‘how many are there?’. More NGS studies are required to answer these questions in a range of populations with differing health status, age and in different geographic locations ideally using a standardized bioinformatics pipeline. This will provide the foundation for much-needed translational work, potentially indicating a ‘universal’ oral medium such as saliva to facilitate larger studies (as sample collection is easy). A comprehensive map of the oral resistome will focus work on ARGs, which pose the most significant threat to human health the genes that can mobilize [39]. Metagenomic platforms including the state-of-the-art long-read sequencing provide association analysis between ARGs and species. This information is crucial as it may reveal the effects of AMR on bacterial physiology, which is poorly understood at present. This knowledge may provide alternate therapeutic pathways such as altering bacterial metabolism to improve the efficacy of current antibiotics, rather than simply relying on the future development of new antibiotics [40].

## supplementary material

10.1099/jmm.0.001866 Uncited Fig. S1.

10.1099/jmm.0.001866 Uncited Table S1.

## Abbreviations

AMR: antimicrobial resistance
ARG: antimicrobial resistance gene
CARD: Comprehensive Antimicrobial Resistance Database
MDR: multidrug resistance
MLS: macrolide/lincosamide/streptogramin
NGS: next-generation sequencing
RCS: root canal system
