# Supplementary material for: Mapping the oral resistome: a systematic review
Source: J Med Microbiol. 2024 Aug 12;73(8):001866. doi: 10.1099/jmm.0.001866 (PMC11318793; doi:10.1099/jmm.0.001866)
Supplement: Uncited Fig. S1. [file jmm-73-01866-s001.pdf]

Supplemental Figure

Database searches undertaken for this review a) Medline, b) CINAHL. C) Embase, d) Scopus and e) Web of Science. The time period for this review was restricted, from 2015 – August 2023.

a) DATABASE: Medline (OVIDSP) – Coverage 1946- Present

1	Drug Resistance, Microbial/ Or Drug Resistance, Bacterial/
2	((Antibiotic-Resistan* Or Antibiotic Resistan*) Adj3 (Gene* Or Bacteria)).mp.
3	((Antimicrobial-Resistan* Or Antimicrobial Resistan*) Adj3 (Gene* Or Bacteria)).mp.
4	((Antimicrobial Or Antibiotic Or Oral Or Bacterial) Adj3 Resistome).mp.
5	(Microbial Drug Resistan* Or Bacterial Drug Resistan*Resistan* Gene*).mp.
6	Args.Tw.
7	1 Or 2 Or 3 Or 4 Or 5 Or 6
8	Mouth/
9	(Mouth* Or Oral Cavit*).mp.
10	8 Or 9
11	7 And 1
12	Limit 11 To (English Language And Humans And Yr="2015 - 2023")

b) DATABASE: CINAHL Via EBSCO Host – 1982 Present

S1	(MH "Drug Resistance, Microbial+")
S2	"Microbial Drug Resistan*" OR "Bacterial Drug Resistan*" OR "Resistan* Gene*"
S3	((Antibiotic-Resistan* OR Antibiotic Resistan*) N3 (Gene* OR Bacteria))
S4	((Antimicrobial-Resistan* OR Antimicrobial Resistan*) N3 (Gene* OR Bacteria))
S5	((Antimicrobial OR Antibiotic OR Oral OR Bacterial) N3 Resistome)
S6	TI Args OR AB Args
S6	S1 OR S2 OR S3 OR S4 OR S5 OR S6
S7	(MH "Mouth+")
S8	Mouth* Or "Oral Cavit*"
S9	S7 OR S8
S10	S6 AND S

c) DATABASE: Embase (OVID-SP) – Coverage 1947- Present

1 (Microbial Drug Resistan* OR Bacterial Drug Resistan* OR Resistan* Gene*).mp

2 ((Antibiotic-Resistan* Or Antibiotic Resistan*) Adj3 (Gene* Or Bacteria)).mp

3 ((Antimicrobial-Resistan* Or Antimicrobial Resistan*) Adj3 (Gene* Or Bacteria)).mp

4 ((Antimicrobial Or Antibiotic Or Oral Or Bacterial) Adj3 Resistome).mp

5 Args.Tw. 4720

7 1 Or 2 Or 3 Or 4 Or 5

8 Mouth/

9 (Mouth* Or Oral Cavit*).mp

10 8 Or 9

11 7 And 10

12 Limit 11 To (Human And English Language And Yr="2015 - 2023")

d) DATABASE: Scopus Via Elsevier Coverage-1996 To Present

(TITLE-ABS-KEY ("Microbial Drug Resistan*" OR "Bacterial Drug Resistan*" OR "Resistan* Gene*" OR Args) OR TITLE-ABS-KEY ("Antibiotic-Resistan*" OR "Antibiotic Resistan*" W/3 Gene* OR Bacteria) OR TITLE-ABS-KEY ("Antimicrobial-Resistan*" OR "Antimicrobial Resistan*" W/3 Gene* OR Bacteria) OR TITLE-ABS-KEY (Antimicrobial OR Antibiotic OR Oral OR Bacterial W/3 Resistome) AND TITLE-ABS-KEY (Mouth* OR "Oral Cavit*") AND TITLE-ABS-KEY (Human*)) AND PUBYEAR > 2015 AND PUBYEAR < 2023 AND (LIMIT-TO (LANGUAGE , "English"))

e) DATABASE: Web Of Science Via Clarivate – Coverage 1900 To Present

#1 "Microbial Drug Resistan*" OR "Bacterial Drug Resistan*" OR "Resistan* Gene*" (Topic)

#2 (("Antibiotic-Resistan*" OR "Antibiotic Resistan*") NEAR/3 (Gene* OR Bacteria)) (Topic)

#3 (("Antimicrobial-Resistan*" OR "Antimicrobial Resistan*") NEAR/3 (Gene* OR Bacteria)) (Topic)

#4 ((Antimicrobial OR Antibiotic OR Oral OR Bacterial) NEAR/3 Resistome) (Topic)

#5 #1 OR #2 OR #3 OR #4

#6 Mouth* OR "Oral Cavit*" (Topic)

#7 #5 AND #6

#8 #5 AND #6 And 2015 Or 2016 Or 2017 Or 2018 Or 2019 Or 2020 Or 2021 Or 2023 Or 2022 (Publication Years) And English (Languages)
